# Spatio-Temporal Characteristics of Inhibition Mapped by Optical Stimulation in Mouse Olfactory Bulb

**DOI:** 10.3389/fncir.2016.00015

**Published:** 2016-03-22

**Authors:** Alexander Lehmann, Anna D’Errico, Martin Vogel, Hartwig Spors

**Affiliations:** ^1^Max Planck Institute of BiophysicsFrankfurt am Main, Germany; ^2^Department of Neuropediatrics, Justus-Liebig-UniversityGiessen, Germany

**Keywords:** olfactory bulb, inhibitory interactions, spatiotemporal patterns, optogenetics, *in vivo*, channel rhodopsin

## Abstract

Mitral and tufted cells (MTCs) of the mammalian olfactory bulb are connected via dendrodendritic synapses with inhibitory interneurons in the external plexiform layer. The range, spatial layout, and temporal properties of inhibitory interactions between MTCs mediated by inhibitory interneurons remain unclear. Therefore, we tested for inhibitory interactions using an optogenetic approach. We optically stimulated MTCs expressing channelrhodopsin-2 in transgenic mice, while recording from individual MTCs in juxtacellular or whole-cell configuration *in vivo*. We used a spatial noise stimulus for mapping interactions between MTCs belonging to different glomeruli in the dorsal bulb. Analyzing firing responses of MTCs to the stimulus, we did not find robust lateral inhibitory effects that were spatially specific. However, analysis of sub-threshold changes in the membrane potential revealed evidence for inhibitory interactions between MTCs that belong to different glomerular units. These lateral inhibitory effects were short-lived and spatially specific. MTC response maps showed hyperpolarizing effects radially extending over more than five glomerular diameters. The inhibitory maps exhibited non-symmetrical yet distance-dependent characteristics.

## Introduction

Neurons in sensory systems are often spatially arranged such that cells responding to similar stimuli cluster in functional or even morphologically defined modules. In the mammalian olfactory bulb (OB) this modular organization is governed by the convergence of axons from olfactory sensory neurons (OSNs) expressing the same receptor type onto the same functional unit, the glomeruli (Mombaerts et al., 1996). Principal OB neurons, the mitral and tufted cells (MTCs), primarily receive sensory input through a single apical dendrite ramifying in only one glomerulus, and they project their axons via the lateral olfactory tract (LOT) to the olfactory cortex (Shepherd et al., 2004).

Lateral interaction between neurons responding to similar stimuli is a general mechanism for contrast enhancement and optimization of sensory representations (Giridhar et al., 2011; Isaacson and Scanziani, 2011). It could serve to de-correlate stimulus representations (Friedrich and Laurent, 2001) and improve olfactory discrimination performance (Abraham et al., 2010). Evidence for lateral inhibition in the OB has been collected for many years (Shepherd et al., 2007; Valley and Firestein, 2008) by anatomical descriptions of the underlying circuitry (Mori et al., 1983; Shepherd et al., 2004; Kim et al., 2012), *in vivo* (Yamamoto, 1961; Phillips et al., 1963, 2012; Yamamoto et al., 1963; Meredith, 1986; Wilson and Leon, 1987; Buonviso and Chaput, 1990; Imamura et al., 1992; Ezeh et al., 1993; Yokoi et al., 1995) and *in vitro* electrophysiological studies (Nowycky et al., 1981; Jahr and Nicoll, 1982; Isaacson and Strowbridge, 1998; Christie et al., 2001; Urban and Sakmann, 2002; Aungst et al., 2003). The OB network provides lateral inhibition on at least two levels: (I) In the glomerular layer ‘short axon’ cells and periglomerular cells provide inhibition between glomeruli (Aungst et al., 2003; Vucinic et al., 2006); (II) in the external plexiform layer (EPL) basal dendrites of MTCs form reciprocal synapses with granule cells (GCs) and parvalbumin-expressing interneurons (PV cells) allowing for lateral and recurrent inhibition (Shepherd et al., 2004; Kato et al., 2013; Miyamichi et al., 2013).

To date the spatio-temporal profile of inhibitory interactions in the OB remains unclear *in vivo*. Mapping these interactions with high temporal precision is crucial because changes of odor representations occur on the timescale of milliseconds (Spors and Grinvald, 2002; Spors et al., 2006; Cury and Uchida, 2010; Shusterman et al., 2011) and odor discrimination can be accomplished within a fraction of a second (Uchida and Mainen, 2003; Abraham et al., 2004; Rinberg et al., 2006a). Furthermore, Fukunaga et al. (2014) have shown that inhibition mediated by GCs is effective in a very short time window most likely shaping MTC spike timing.

In order to map inhibitory connections directly and to resolve the conflicts between earlier studies (Luo and Katz, 2001; Arenkiel et al., 2007; Fantana et al., 2008), we set out to systematically map the input to individual MTCs in the EPL mediated by inhibitory interneurons *in vivo*. In contrast to the use of odorant stimuli, optical stimulation allowed us to control with very high temporal precision the activation of different MTCs distributed over the dorsal OB while recording the response of single MTCs. By direct stimulation of MTCs we focused our observations on effects mediated in the EPL by inhibitory interneurons, since we most likely omitted the lateral inhibitory effects that occur in the glomerular layer after sensory input via OSNs (Shepherd et al., 2004; Fukunaga et al., 2014). We first tested for MTC interactions at the supra-threshold level analyzing the action potential (AP) rate in response to the optical stimulus. Maps of stimulation efficacy contained one excitatory hotspot corresponding to the MTC’s parent glomerulus and only weak specific inhibitory interactions in the surrounding area. However, analysis of sub-threshold activity, i.e., the membrane potential of MTCs, revealed hyperpolarizing interactions in response to stimulation of glomerular units in the surrounding area. We found spatially inhomogeneous, yet distance dependent, inhibitory fields spanning more than five glomerular diameters in 5 out of 14 recorded MTCs, and the time window of the observed lateral inhibitory effects was very short, on a timescale <50 ms.

## Materials and Methods

### Surgical Preparation

Forty one male and female Thy1-ChR2-YFP mice (Stock #7612 The Jackson Laboratory; Arenkiel et al., 2007), 5–12 weeks-old, were used for this study. Mice were bred in a tg/tg colony. Two mice were offspring from a cross with MOL2.3 IGITL mice (Conzelmann et al., 2000), and three mice were offspring from a cross including the OMP-spH knock-in (Bozza et al., 2004). All animal care and experimental procedures were approved by the regional authorities and carried out in accordance with the animal ethics guidelines of the Max Planck Society. Mice were anesthetized using Urethane (1.5 g/kg i.p.). During the initial surgery Isoflurane (0.5–1.5% in oxygen) was supplemented. Urethane was added (10–20% of the initial dose) throughout the experiments based on the depth of anesthesia as assessed by whisking, withdrawal reflex, respiratory rate and heartrate. Using a heating pad and a rectal probe (FHC, Bowdoin, ME, USA) we kept the body temperature between 36.5 and 37.5°C. We monitored and recorded the respiration using a piezoelectric strip (WPI, Sarasota, FL, USA) around the thorax. We attached a metal plate with a central opening to the dorsal skull overlying the OB in order to hold the animal in a stereotactic frame. A cranial window (2 mm × 1.5 mm) was opened using a dental drill (Ossada Electric, Tokyo, Japan). Pulsation of the OB tissue was reduced by covering the cranial window with agar (1.5% in artificial cerebral spinal fluid) and by opening the cisterna magna.

### Electrical Recording and Data Acquisition

For juxtacellular recordings of MTCs we used low-resistance borosilicate patch pipettes (5–7 MΩ, OD 2.0 mm, ID 1.0 mm, Hilgenberg, Malsfeld, Germany) pulled on a DMZ puller (Zeitz Instruments, Munich, Germany). Pipettes were filled with standard artificial cerebral spinal fluid. We searched MTCs by 2 μm stepwise advancement of the electrode in voltage clamp mode, while monitoring the electrode resistance. Upon an increase of the electrode resistance the exposed OB surface was flashed for 20 ms (central wavelength 460 nm, intensity at sample 6 mW/mm^2^). This confirmed that a neuronal element was causing the resistance increase and that the neuron could be driven by optical stimulation with a short latency. For whole-cell recordings we used the same pipettes and recording system as for the juxtacellular configuration. To achieve the whole-cell patch clamp configuration we followed the standard protocol described by Margrie et al. (2002). Our intrapipette solution contained, in mM: K-gluconate 135, HEPES 10, phosphocreatine-Na 10, KCl 4, MgATP 4, GTP 0.3 and biocytin 0–2%, pH 7.2. The junction potential was not subtracted. Data were acquired using an Axoclamp 900A amplifier (Molecular Devices), low pass filtered at 10 kHz and recorded simultaneously on two analog digital recording systems. Sweep based data were acquired at a rate of 20 kHz using a NI PCI-6251 board (National Instruments, Austin, TX, USA) driven by custom written software in the Matlab programming environment. A continuous data stream was simultaneously recorded using an Axon Digidata 1440 board and the PClamp10 software (Axon Instruments & Molecular Devices, Sunnyvale, CA, USA) for the majority of experiments.

### Optical Setup

The dorsal OB was visualized using a Movable Objective Microscope (Sutter, Novato, CA, USA), a Zeiss water immersion lens (10x, N.A. 0.3) and an AxioCam camera (Zeiss, Göttingen, Germany). Care was taken not to expose the OB to strong light. Similar to studies performed *in vitro* (Boucsein et al., 2005; Wang et al., 2007; Jerome et al., 2011; Liang et al., 2011), in brain explant preparations (Blumhagen et al., 2011), and *in vivo* (Davison and Ehlers, 2011), we aimed to generate patterns of neuronal activity by optical stimulation. In order to project patterns of light onto the exposed surface of the OB, we coupled a digital mirror device (DMD, 0.7’ XGA, Texas Instruments, Dallas, TX, USA) controlled by a Discovery 1001 board and an ALP2 board (Vialux, Chemnitz, Germany) into the light path using an on axis light engine design and a high power LED (central wavelength 460 nm, Vialux). At full power this setup resulted in a light intensity on the OB surface of 6 mW/mm^2^. The field of view was 2 mm^2^. The ALP2 interface allowed for real-time triggering and a frame rate of up to 8 kHz with a resolution of 1024 × 768 pixels. Each DMD pixel covered an area of 1.6 μm × 1.6 μm on the brain. Using a photodiode the change of average light intensity over the total field of view was recorded simultaneously with the electrophysiological data. This allowed for *post hoc* control of the alignment of the optical stimulation and the neuronal activity. Optical stimulation was controlled using custom written software in the Matlab programming environment (MathWorks, Natick, MA, USA).

#### Dense Noise Stimulation

**FIGURE 1:**
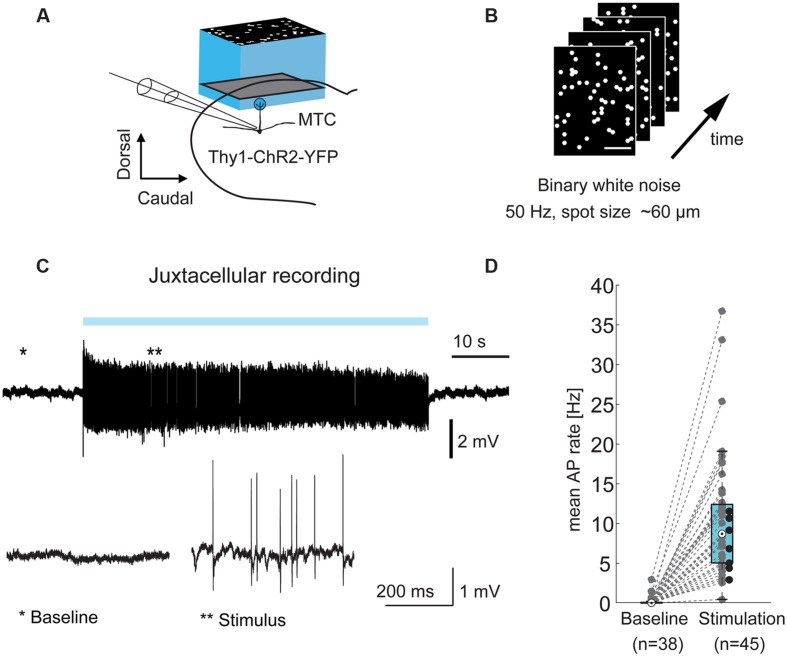
**Optical stimulation drives MTCs. (A)** Experimental setup: olfactory bulb (OB) mitral and tufted cells (MTCs) expressing ChR2 (Thy1-ChR2-YFP) were stimulated using a projection system (digital mirror device, blue LED, central wavelength ∼460 nm). *Left*, MTCs were recorded in juxtacellular or whole-cell configuration during light-stimulation of the dorsal OB. **(B)** Frames from a stimulation movie (3,000 frames) with binary dense white noise; on-probability for individual spots in any given frame is 10%. **(C)** *Top*, example trace from a juxtacellular recording of a MTC during 1 min dense noise stimulation. *Blue bar*, stimulus; *bottom*, zoom-in on highlighted periods; **(D)** AP rate without (baseline, n = 38) and during optical stimulation (n = 45 MTCs), recorded in juxtacellular or whole-cell configuration. Black dots correspond to seven cells without sufficient recorded baseline.

The entire surface of the DMD was tiled with hexagons with a size of 64 μm (diameter of inner circle) unless noted otherwise. Only spots that were projected onto the exposed OB surface were included in the analysis of the whole-cell data. Binary white noise movies were generated such that each spot was active for 10% of the stimulation time. We allowed the number of active spots to vary between frames. The dense stimulus movies were played in 1 min epochs at a rate of 50 Hz. Movie epochs were spaced by 1 min pauses except for three experiments in juxtacellular configuration where pauses ranged from 25 to 40 s and seven experiments in juxtacellular configuration without baseline recorded (plotted separately at the respective locations in **Figures 1** and **3**).

#### Center-Surround Stimulation

In four whole-cell experiments we generated a neuron specific stimulation pattern: a disk (50 μm radius) and a ring (50 or 95 μm inner radius, outer radius including the most distant spot in the field of view, **Figure 11**) were centered on the stimulation hotspot. Intensity of the inner disk was 6 mW/mm^2^. Intensity of the surrounding ring varied from 0 to 0.5 mW/mm^2^. Stimulation was triggered on the respiration cycle and lasted 100 ms.

#### Visualization of Glomeruli *In vivo*

SynaptopHluorin labeled OB glomeruli were imaged using a 2-photon laser scanning microscope (Prairie Technologies, Middleton, TN, USA), a 16x water immersion objective (N.A. 0.8, Nikon) and a MaiTai DeepSee laser (50–170 mW, tuned to 880 nm, 80 MHz repetition rate of pulses 120 fs in length; Spectra-Physics/Newport, Santa Clara, CA, USA). Images (512 × 512 pixels) were acquired at 5 μm steps in the z-direction.

### Data Analysis

#### Spike Detection, Spike Sorting, and Removal of APs from Intracellular Recordings

Data from extracellular recordings were separated oﬄine into a LFP band and an AP band using a 200 Hz non phase-shifting digital filter (6th order Butterworth, Matlab). APs were detected and sorted oﬄine using either a threshold and template based algorithm or the OSort software (Rutishauser et al., 2006). Only data sets with all inter-spike-intervals longer than 2 ms were included. APs were removed from the whole-cell recordings by cutting out a time window of 3–3.25 ms around the peak of the AP, with subsequent linear interpolation and low-pass filtering at 200 Hz. LFP and membrane potential power spectra were calculated after linear de-trending of the data.

#### Forward Correlation

In order to determine the effect of the stimulation on AP rate and the membrane potential with millisecond temporal resolution we calculated the first order forward correlation. To this end, we generated a peristimulus time histogram (PSTH) for each spot triggering on the time points when the respective spot was flashed. For the whole-cell recordings we calculated the average membrane potential triggered on all flashes of each spot after removing the APs (see above). To subtract the non-specific general modulation of the AP frequency or the membrane potential, we generated a PSTH or averaged membrane potential triggered on all frame transitions. This average trace was subtracted from the PSTHs or the membrane potential averages.

#### Statistical Analysis

We present data from 45 MTCs. 31 MTCs were recorded in juxtacellular configuration, 14 MTCs in the whole-cell patch clamp configuration. Unless otherwise noted we report the median with interquartile range. For non-parametric statistical testing we used the Wilcoxon rank sum test. For testing the uniform distribution of circular data we used the Rayleigh Test (Matlab code by Berens, 2009). To compare the distance distributions of excitatory and inhibitory spots we used the Kolmogorov–Smirnov test.

**FIGURE 2:**
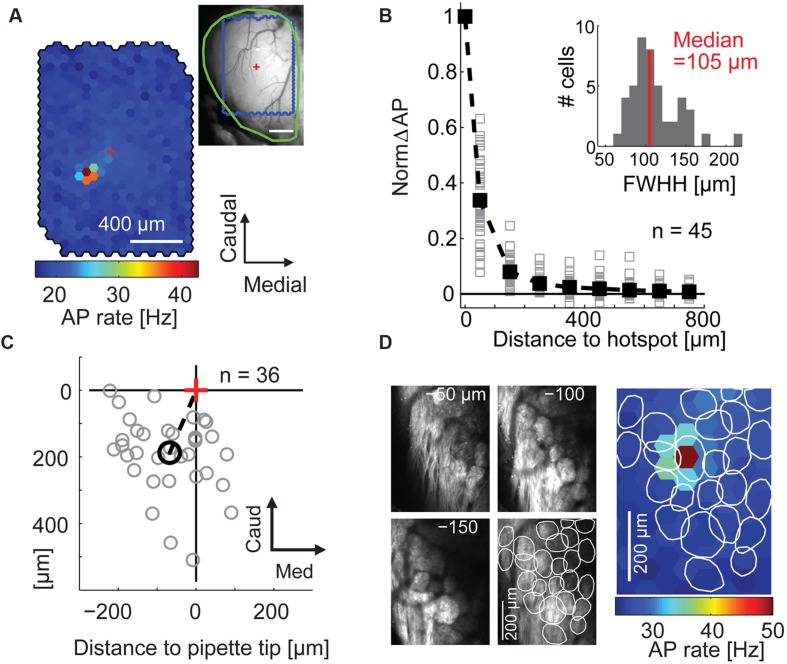
**Maps of stimulation efficacy reveal hotspots that correspond to glomeruli. (A)** Color-coded map of stimulation efficacy: for every stimulated spot the map displays the average AP rate during all frames the spot was flashed; 1,500 flashes per spot. *Top right*, blood vessel pattern of the dorsal OB with the outline of the stimulated area (*blue*), the electrode position (*red cross*), and the outline of the exposed OB (*green*). **(B)** Stimulation efficacy as a function of distance from the hotspot (100 μm bins, n = 45 MTCs, solid squares, average). *Inset*, hotspot width obtained by fitting Gaussians to the activation maps of the individual experiments. *FWHH*, full width at half height. **(C)** Position of the hotspots (*gray circles*) relative to tip of recording electrode (*red cross*) for all 36 MTCs with known pipette tip position. *Black circle*, mean hotspot position. **(D)** Overlay of the color-coded stimulation efficacy and the outlines of glomeruli identified by 2-photon microscopy in the same animal.

As a measure of hotspot tuning width we estimated the full width at half height (FWHH). We fitted a Gaussian function to the AP rate changes at different distances (d) from the hotspot (**Figure 2B**).

$$f\left(x\right)=e^{-\left(\frac{x}{\sigma }\right)2}$$

FWHH was calculated from σ with:

$$FWHH=2*\sigma *\sqrt{\left(\ln\;2\right)}$$

Fitting with one or two additive Gauss terms did not change the FWHH of the first term (data not shown).

To test if the hotspot centers corresponded to the glomerulus outlines we determined the centers of mass for all glomeruli identified using 2-photon laser scanning microscopy (see above). We also calculated the center of mass of the hotspot including the six neighboring hexagons to the spot with the strongest stimulation efficacy. Within the area of outlined glomeruli we calculated the distance of each camera pixel to the closest glomerulus center. For statistical testing we randomly drew 100,000 times one pixel from each of the three experiments with reconstructed glomeruli and averaged their distance values. Finally we compared the average distance of the three hotspot centers to the next glomerulus center (12.9 ± 6.3 μm, mean ± SD) with the distribution of the averaged random distance values (40.6 ± 13.2 μm, mean ± SD).

To quantify the spatial extent of AP rate change (**Figure 5C**) for each MTC we averaged the values of equidistant spots (100 μm bins) with respect to the hotspot. Profiles for the time points (4 ms bins) at which excitatory and inhibitory components were largest across all experiments were normalized to the values of maximal excitation and inhibition, respectively. Finally the median of all 45 normalized profiles was calculated. Confidence intervals (±SEM) of this median profile were determined by bootstrapping 100 times. Control profiles were generated using the 500 shuﬄed data sets.

In order to calculate significance thresholds for forward correlation results and to control for analysis-induced effects we shuﬄed the stimulation time points of individual spots randomly and repeated the analysis above 500 times. Since the analysis was performed for 522 spots in parallel, this provided a distribution of random values with 261,000 entries. To account for the varying AP probability and the varying distribution of membrane potential values within each 20 ms stimulation interval we calculated the 10^-1^ and 10^-3^ percentile (corresponding to one tailed *p*-values of 10^-3^ and 10^-5^) separately for each time bin (1 or 4 ms), depending on the experiment. In the analysis of the spatial distribution of excitatory and inhibitory spots (see **Figure 8B**) we took into account the different amplitudes and signal-to-noise ratios of depolarizing and hyperpolarizing membrane potential changes. For this, we combined two significance criteria: (1) Spots had to pass the threshold calculated using shuﬄed control data, and (2) Spots had to pass a threshold calculated as percentage of the maximal depolarization or the maximal hyperpolarization.

Receiver operating characteristics (ROCs, **Figure 8C**) for the detection of inhibitory spots were calculated for MTCs recorded intracellularly. We plotted the frequency of inhibitory spots during the time window of inhibition (40 ms after stimulus start, 4 ms time bins) versus the frequency of inhibitory spots during the base line period (100 ms before stimulus start, 4 ms time bins) while varying the detection threshold for inhibitory spots, in terms of significance level.

In the experiments with center and surround stimulation, the amount of lateral inhibition (**Figure 11D**) was calculated as the average difference between traces with surround stimulation and traces with center stimulation only in a time window specific for each MTC. This time window started with the end of the stimulus. The duration of the time window was 2x the time to half maximal inhibition, t_half_ (**Figure 11F**). Confidence intervals (mean ± SEM and mean slopes ± SEM) were calculated by bootstrapping 100 times across the 50 repetitions of the same stimulus.

## Results

### Illumination of the Parent Glomerulus Specifically Drives Mitral and Tufted Cells

Mitral and tufted cells typically receive sensory input from a single glomerulus (parent glomerulus). In the transgenic mouse line used in this study, MTCs express ChR2 from the Thy1 promoter and can be stimulated by illumination of the dorsal OB surface (Arenkiel et al., 2007). First, we asked if it is possible to record from a given MTC and map the location of its parent glomerulus by illuminating the dorsal OB using binary white noise stimuli with 50 Hz frame transitions (**Figures 1A,B**). The blue light patterns consisted of hexagons with an inner circle diameter of 64 μm. Every single hexagon, from now also termed “spot,” had an on-probability of 10%. During mapping we recorded from individual MTCs in juxtacellular (**Figure 1C**) or whole-cell patch clamp configuration. The optical stimulation increased the average AP rate of MTCs from 0.0 Hz at baseline (median, interquartile range: 0.0–0.1 Hz, *n* = 38) to 8.7 Hz during stimulation periods (median, interquartile range: 5.0–12.4 Hz, *n* = 45, *p* = 8^∗^10^-15^, Wilcoxon rank sum test, **Figure 1D**).

Calculating the stimulation efficacy for the individual hexagons (**Figure 2A**) we obtained maps with clear hotspots (i.e., spots at which stimulation leads to an increase in AP rate) in 45 out of 59 neurons (representative example in **Figure 2A**). Only data of these 45 neurons (31 juxtacellular and 14 whole cell individual recordings) were further analyzed. With increasing distance from the hotspot, the stimulation efficacy dropped sharply with a median FWHH of 105 μm (interquartile range: 92–128 μm, **Figure 2B**). Hotspots were generally shifted relative to the electrode tip (rostral 188 μm ± 111 μm, lateral 68 μm ± 90 μm, mean ± SD, *n* = 36 neurons, **Figure 2C**); this shift corresponds to the tilt of MTC apical dendrites (Buonviso et al., 1991) suggesting that we drove MTCs from their parent glomeruli. To confirm that the hotspots corresponded to individual glomeruli we used 2-photon microscopy to image glomeruli in three mice expressing synaptopHluorin in all sensory neurons (OMP-spH) and ChR2 from the Thy1 promoter. In all three examples the hotspot matched the position of a morphologically identified glomerulus (representative example in **Figure 2D**) and the hotspot centers were significantly closer to the nearest glomerulus center than expected by chance (*p* = 0.002, *n* = 3).

Taken together these data indicate that we were driving MTCs mainly from their apical dendrite and tuft within their parent glomerulus. Thus, by illuminating an area the size of a single glomerulus, we could drive MTCs with high specificity.

### Stimulus Imposed Oscillation of LFP and AP Rate

**FIGURE 3:**
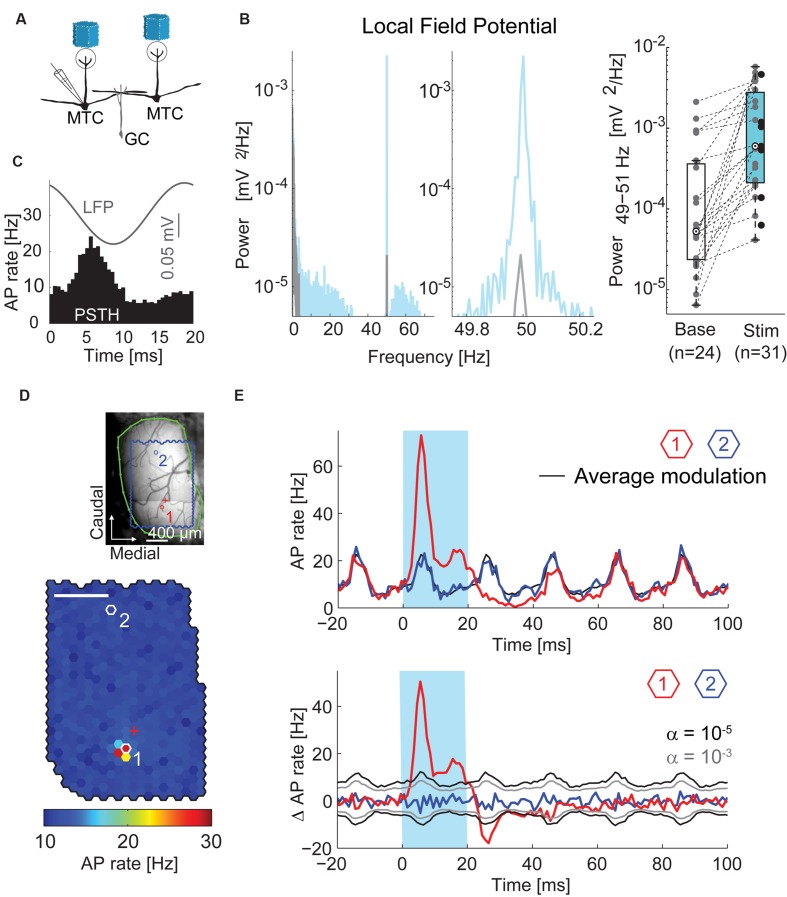
**Oscillations and time courses of AP rate. (A)** Simplified diagram of the MTC/granule cell (GC) circuitry and experimental setup. **(B)** *Left*, local field potential (LFP) power spectral density of a single recording before (*gray*) and during (*blue*) stimulation; *middle*, zoom-in around stimulation frequency; *right*, boxplot of LFP modulation (49–51 Hz) before and during stimulation for all recorded MTCs. **(C)** Average LFP (*gray*) and peristimulus time histogram (PSTH, *black*) of a single recording triggered on all frame transitions (0.5 ms time bins, 30,000 frames). **(D)** Color-coded map of stimulation efficacy and blood vessel pattern (as in **Figure 2A**) of a different MTC. *Red* (1) and *blue* (2) *hexagons* indicate spots with PSTHs in **(E)**; *red cross*, electrode position. Scale bar 400 μm. **(E)** *Top*, PSTH (1 ms time bins, 3,000 stimuli) triggered on the onset of the 20 ms stimuli (*blue box*). *Red*, spot with maximal efficacy (hotspot); *black*, average AP rate modulation; *blue*, spot without change in AP rate. See **(D)** for spot locations. *Bottom*, same data after subtracting the average AP rate modulation (black line on the top plot). *Gray*, significance levels calculated using shuﬄed data.

The optical stimulation with frame transitions at 50 Hz evoked prominent oscillations of the local field potential (LFP, **Figures 3B,C**). Accordingly, during stimulation periods the LFP power spectra of single recordings contained sharp peaks at stimulation frequency (**Figure 3B**). The LFP-power at 50 Hz increased from 5.3^∗^10^-5^ mV^2^/Hz (median, interquartile range: 2.4^∗^10^-5^ to 3.6^∗^10^-4^ mV^2^/Hz, *n* = 24 MTC) before stimulation to 6.0^∗^10^-4^ mV^2^/Hz (median, interquartile range: 2.1^∗^10^-4^ to 2.8^∗^10^-3^ mV^2^/Hz, *n* = 31 MTCs, *p* = 5^∗^10^-5^ Wilcoxon rank sum test, **Figure 3B**) during stimulation. Averaging the LFP responses and calculating PSTHs from the AP responses to all stimulation frames (with a length of 20 ms each) confirmed the stimulus-locked temporal modulation of neuronal activity (representative recording in **Figure 3C**). The observed oscillatory AP rate modulation was significant in 29 out of 45 MTCs (Rayleigh test, *p* < 0.05). Presumably, the rhythmicity of the optical dense noise stimulus (activating many MTCs simultaneously) and synaptic interactions within the OB circuitry generated the oscillatory activity and locked the LFP and AP activity tightly to the frame transition frequency. Changing the stimulation frequency around 50 Hz also changed the frequency of the LFP (data not shown). In the following analysis of MTC responses, we aimed to reveal effects of stimulating single spots, not the global effects of the whole stimulus pattern. Therefore, we subtracted the average modulation of the AP rate, i.e., the average response to all stimulus frames, from the AP rate changes elicited by flashing single spots (**Figure 3E**).

### Inhibition Follows Excitation from the Hotspot

**FIGURE 4:**
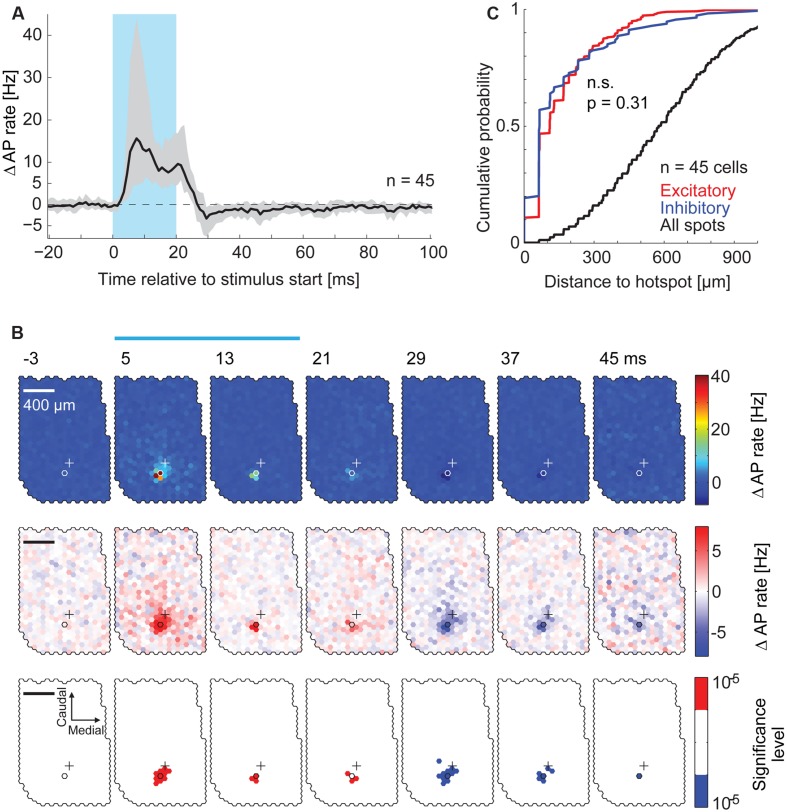
**Spatial distribution of inhibitory effects on AP rate. (A)** PSTHs at the hotspots pooled across all recorded MTCs (n = 45). The median PSTH (*black*) shows an increase in AP rate during the stimulus and a reduction after the end of the stimulus. *Gray area*, interquartile range; *blue box*, stimulus. **(B)** Data from the same MTC as in **Figure 3D**. PSTHs (4 ms time bins, 3,000 stimuli) of all spots were color-coded. Every second time bin is displayed; start of the time bin is in ms; *blue bar*, stimulus; *outlined hexagons*, hot spot; *crosses*, pipette tip position. The average AP rate modulation (black trace in **Figure 3E**) is subtracted. *Top*, color map spanning the entire range of data. *Middle*, color map with increased contrast. *Bottom*, spots above (*red*) or below (*blue*) a significance threshold of α = 10^-5^ calculated using shuﬄed control data. Scale bar 400 μm. **(C)** Cumulative probability of the distance from the hotspot of significant excitatory spots (*red*, n = 376), significant inhibitory spots (*blue*, n = 114), and all spots (*black*, n = 23,490) for all 45 MTCs (p = 0.31, KS-test). Significant spots occurring in multiple time bins (4 ms) were only counted once.

Stimulation of the hotspot (e.g., **Figure 3D**) resulted in an increase in AP rate of of 21.7 Hz (median, interquartile range: 11.4–52.4, *n* = 45, calculation in 1 ms time bins, representative hotspot in **Figure 3E**, *red trace*). As described above, this increase in firing was restricted to the hotspots. Stimulation of remote spots did not lead to an increase in AP rate (e.g., **Figures 3D,E**, *blue trace*). Following the end of the hotspot stimulation, the AP rate decreased and dropped below the average AP rate (individual example: **Figure 3E**, pooled data: **Figure 4A**). In 32 of 45 cells the AP rate significantly decreased below baseline about 8 ms after the end of hotspot stimulation (median, interquartile range: 7–9.5 ms, *n* = 32, α = 10^-5^). Cells that did not show this decrease in AP rate generally had lower firing rates than other MTCs. As a measure of the amount of post-stimulus AP rate reduction, we calculated the mean AP rate change in a window of 8–100 ms after stimulus end (**Figure 4A**). The post-stimulus changes significantly correlated with the overall firing activity of the cells during stimulation (*r* = 0.76, *p* < 10^-8^). These findings are well in line with the description of recurrent inhibition and inhibition due to sister MTCs belonging to the same glomerulus (Urban and Sakmann, 2002). They are also consistent with the notion that both effective AP propagation along lateral dendrites and recurrent inhibition increase with the number of APs fired (Margrie et al., 2001). However, intrinsic conductances and relative refractoriness of the MTCs themselves could have contributed to this AP rate reduction. Also, a floor-effect might be an explanation, since MTCs with low baseline activity can *per se* only have small reductions in AP rate. As the relative contribution of the different mechanisms of AP rate reduction was not determined here, we call it from here on ‘delayed self-inhibition.’

### Distance Dependent Effects on AP Rate

We asked if stimulation of MTCs belonging to neighboring or more distant glomeruli also affected the firing rate of a given MTC. To analyze the effects resulting from stimulation of remote spots we calculated PSTHs in 4 ms time bins for every single spot on the exposed OB surface. Again, the average AP rate modulation was subtracted. To visualize the contribution of all the spots to firing rate changes, we displayed these PSTHs jointly as a movie using false colors (representative example in **Figure 4B**). Increasing the color-contrast in the ‘movie’ (**Figure 4B**), the delayed self-inhibition described above became more obvious. However, we did not detect significant (α = 10^-5^) inhibitory spots in the surround. The distance distribution of excitatory and inhibitory spots detected in the maps from all recordings did not differ (**Figure 4C**, *n* = 45, *p* = 0.31, Kolmogorov–Smirnov test). Likewise, 80% of significant inhibitory spots were within 200 μm from the hotspot. From this spatial restriction of excitatory and inhibitory spots we argued initially that both the increase in AP rate of a given MTC and the following delayed self-inhibition were a result of stimulating MTCs belonging to the same parent glomerulus.

**FIGURE 5:**
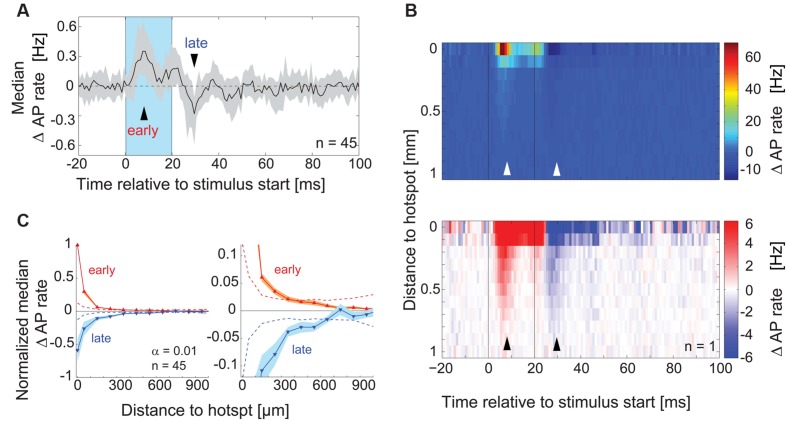
**Lateral inhibitory effects on AP rate. (A)** For each MTC the median across PSTHs of all spots was calculated. The plot shows the median (*black line*) and interquartile range (*gray area*) of these median PSTHs across all MTCs (n = 45). *Blue box*, stimulus; *arrowheads*, time points of maximal AP rate increase and decrease respectively. **(B)** Color-coded average AP rate change as a function of time (x-axis, 1 ms bins) and distance from the hotspot (y-axis, 100 μm bins) of an individual experiment. *Top*, color map spanning the entire range of data; *bottom*, color map with increased contrast. *Black lines*, begin and end of the stimulus; *arrowheads* correspond to time points in **(A)**. **(C)** Change of the AP rate at the time of maximal excitation ‘early’ (*red trace*, 9–12 ms post-stimulus start) and at the time of maximal inhibition ‘late’ (*blue trace*, 9–12 ms post-stimulus end) as a function of distance from the hotspot (100 μm bins). Data from each MTC were normalized to the values of maximal excitation and inhibition, respectively. *Left panel*, whole data range; *right panel* enlarged view. *Solid lines*, median of all 45 MTCs. *Broken lines*, significance levels (α = 0.01) calculated using shuﬄed data.

To rule out the possibility that we did not detect subtle inhibitory effects that might be caused by stimulation of the surrounding area, we analyzed the time course of overall excitatory and inhibitory components in the responses pooling data across equidistant spots (**Figure 5B**) and across all recordings (**Figures 5A,C**, *n* = 45). We calculated the median AP rate change at a given time point across all spots, across all the cells recorded (**Figure 5A**, 1 ms bins): consistent with the previous analysis, the median AP rate change increased shortly after stimulation onset (‘early’ in **Figure 5A**) and decreased below baseline within 10 ms after the end of stimulation (‘late’). To better examine the distance and time dependence of the inhibitory effects, we plotted the color coded AP rate change as a function of distance from the hotspot (100 μm bins, y-axis), and time relative to the stimulus onset (1 ms bins, x-axis). This visualization (representative example in **Figure 5B**) allowed the detection of two inhibitory components: (1) Delayed self-inhibition following stimulation at the hotspot, and (2) a reduction of the AP rate by stimulation even at distances more than 500 μm from the hotspot (5–12 ms after the stimulus end).

We tested for significance of this AP rate reduction in the surrounding area by pooling data from all experiments (*n* = 45) at the time point of maximal inhibition (**Figure 5A**, ‘late’): for every spot, the data in this single time bin were normalized with respect to the minimal value in this time bin. We then averaged equidistant spots relative to the hotspot (100 μm bins), which gave us a normalized spatial profile of the inhibitory effects with the hotspot in its center (**Figure 5C**). The observed AP rate reduction was significant up to 550 μm from the hotspot (α = 0.01, 9–12 ms post-stimulus, *n* = 45, **Figure 5C**). Excitatory effects, determined at the time point of maximal excitation (‘early’), also extended several μm away from the hotspots. However, the normalized excitatory effects were only significant up to a distance of 250 μm from the hotspot (**Figure 5C**) despite the generally bigger excitatory amplitude. These excitatory effects might be explained by subtle stimulation of MTC apical and lateral dendrites that lie close to the OB surface (compare to excitatory field in **Figure 4B**, *middle*). However, the inhibitory effects were also significant at larger distances, over 250 μm away from the hotspot. The further spread of inhibitory compared to excitatory effects suggested that we were not simply observing a self-inhibitory effect stemming from the preceding stimulation of the recorded MTC.

In summary, juxtacellular recordings from MTCs provided evidence for a delayed self-inhibition after stimulation of the parent glomerulus. Moreover, by pooling data from many neurons, we were able to detect subtle but significant inhibitory effects by stimulating distant (>500 μm) glomerular units. However, by analyzing AP responses, we could not resolve potential inhibitory effects that were elicited by the stimulation of single spots in the surrounding area of a given MTC. Thus for the analysis of specific interactions between MTCs a more sensitive recording technique or substantially longer recordings are required.

### Whole-Cell Recordings Reveal Distance-Dependent Inhibition

**FIGURE 6:**
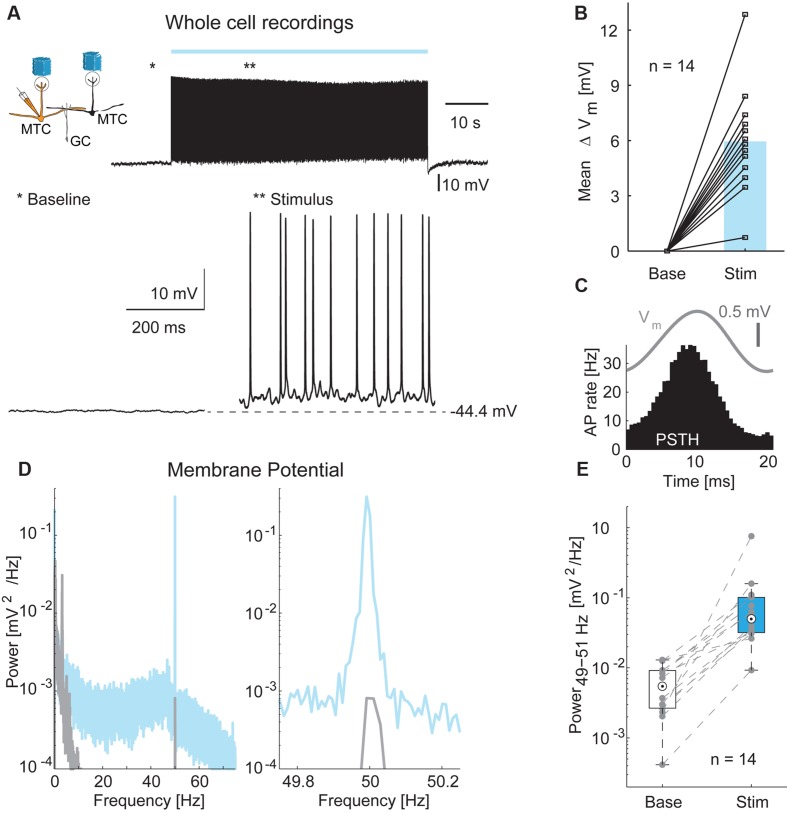
**Membrane potential changes during optical stimulation. (A)** *Top left*: Simplified diagram of the probed MTC/GC circuitry. *Right*: Example trace from a whole-cell recording of a MTC during 1 min stimulation. *Bottom*: Zoom-in on baseline period and stimulation period indicated by asterisks. **(B)** Membrane potential depolarization during stimulation compared to baseline without optical stimulation. *Blue bar*, median, n = 14 whole-cell recordings. **(C)** Average membrane potential (V_m_, *gray*) and PSTH (*black*) of a single cell triggered on all frame transitions (0.5 ms time bins, 30,000 frames). **(D)** Membrane potential power spectral density of a single cell before (*gray*) and during (*blue*) optical stimulation. *Right*, zoom-in on γ-frequency around 50 Hz. **(E)** Membrane potential modulation (49–51 Hz) before and during optical stimulation for all 14 MTCs recorded intracellularly.

In order to measure inhibitory input on MTCs more directly, we performed whole-cell current-clamp recordings in 14 MTCs (**Figure 6**) and analyzed membrane potential changes resulting from optical stimulation. Recorded membrane potential traces were analyzed after removing APs. The optical stimulus resulted in a median membrane potential depolarization of 5.9 mV (median, interquartile range: 4.5–6.9 mV, **Figure 6B**, *n* = 14). As observed for LFP and AP rate, a 50 Hz oscillation was also present in the subthreshold membrane potential modulation (representative example **Figures 6A,C–E**). This oscillation was absent or much weaker during periods without stimulation (**Figure 6E**, median power spectral density of 0.005 mV^2^/Hz, interquartile range: 0.003–0.009 mV^2^/Hz at baseline vs. 0.050 mV^2^/Hz, interquartile range: 0.032–0.102 mV^2^/Hz during stimulation periods, *p* < 10^-4^, *n* = 14).

**FIGURE 7:**
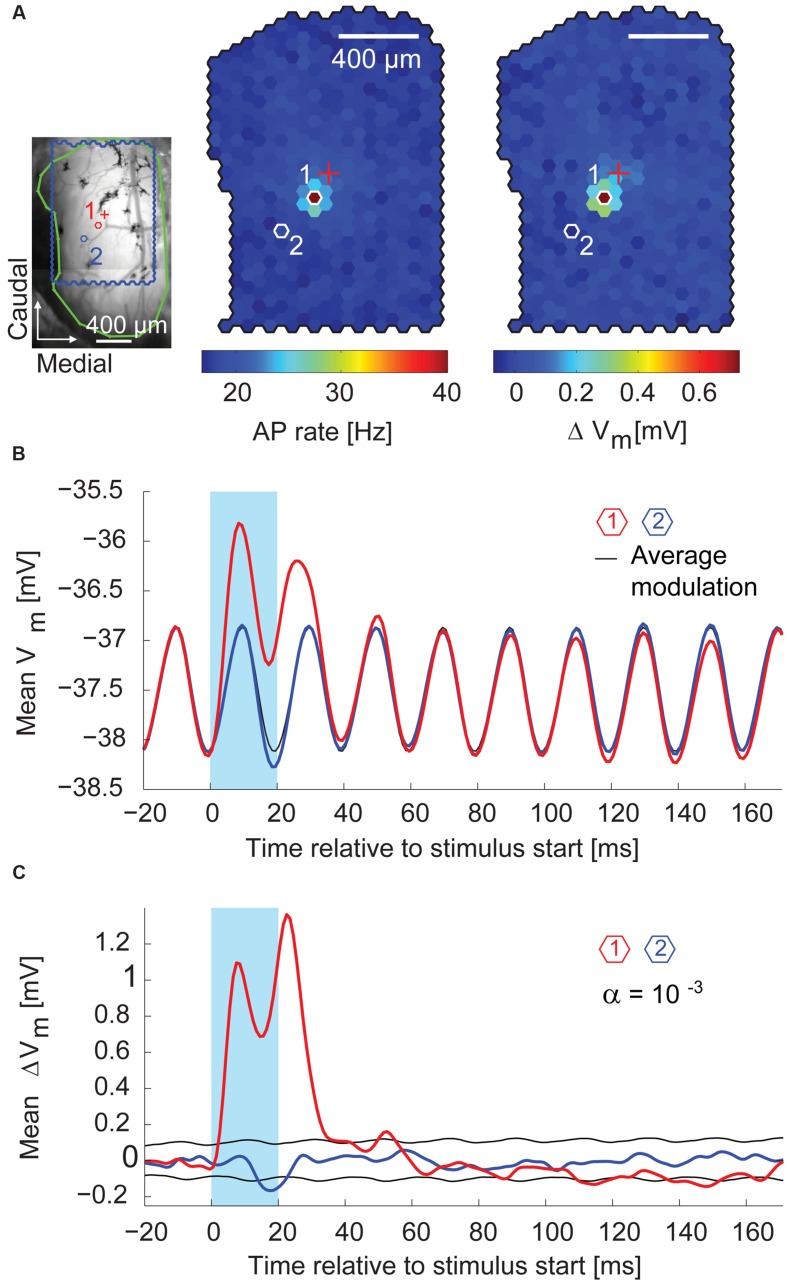
**Excitatory and inhibitory effects on the membrane potential. (A)** *Left*, blood vessel pattern of the dorsal OB with the outline of the stimulated area (*blue*), electrode position (*red cross*), and outline of the exposed OB (*green*). *Red (1)* and *blue (2) hexagons*, positions of spots with membrane potential time course in **(B)**. *Middle*, color-coded map of stimulation efficacy calculated as average AP rate during the 20 ms flash of the individual spots; 3,000 flashes per spot. *Right*, color-coded map of average membrane potential change during the 20 ms flash of the individual spots. Scale bar 400 μm. **(B)** Average membrane potential (1 ms time bins, 3,000 flashes per spot) triggered on the onset of the 20 ms stimuli (*blue box*). *Red*, stimulation spot (1) with maximal depolarization (hotspot); *black*, average membrane potential modulation; *blue*, spot (2) close to the hotspot. See **(A)** for spot location. **(C)** Same data as in **(B)** after subtracting the average membrane potential modulation (*black* line in **B**). *Black*, significance level calculated using shuﬄed data.

During the first 10–30 ms after the stimulus start, the stimulus spot that evoked most APs (the hotspot) also induced the strongest depolarization on top of the membrane potential oscillation (median depolarization of 0.63 mV, interquartile range: 0.44–0.83 mV, *n* = 14 MTCs). **Figure 7A** shows, for a representative recording, the superimposition of the AP hotspot with the membrane potential hotspot. Shortly after the end of the stimulus, the membrane potential at the hotspot returned to pre-stimulus values (**Figure 7B**, *red trace*). In comparison to the hotspot, the specific depolarization was very weak at a nearby spot (**Figure 7B**,*blue trace*). However, by flashing this neighboring spot the membrane potential was significantly hyperpolarized (**Figure 7B**, *middle*). It is noteworthy that stimulating at this location did not elicit a depolarization of the membrane potential preceding the hyperpolarization. This finding suggests a distinct form of hyperpolarization which is not mediated by recurrent or delayed self-inhibition. These first observations indicated that whole-cell recordings allowed not only the detection of excitatory but also of inhibitory inputs, and that these inhibitory effects could be attributed to the stimulation of single spots around the parent glomerulus of a given MTC.

**FIGURE 8:**
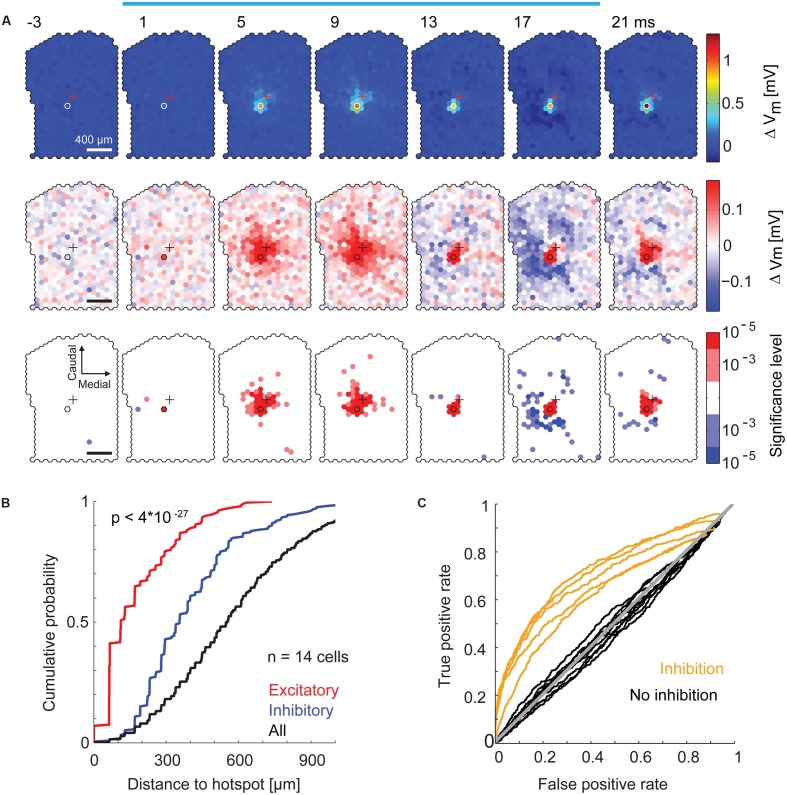
**Specific inhibitory effects on the membrane potential by surround stimulation. (A)** Data from the same MTC as in **Figure 7**. Color-coded average membrane potential (4 ms time bins, 3,000 stimuli) for all spots. Start of the time bin is indicated in ms; *blue bar*, stimulus; *outlined hexagons*, hot spot; *crosses*, pipette tip position. The average membrane potential modulation (black trace in **Figure 7B**) was subtracted. *Top*, color map spanning the entire range of data. *Middle*, color map with increased contrast. *Bottom*, spots above (*red*) or below (*blue*) a significance threshold of α = 10^-3^ and α = 10^-5^ calculated using shuﬄed control data. Scale bar 400 μm. **(B)** Cumulative probability of the distance from the hotspot of significant excitatory spots (*red*, n = 199), significant inhibitory spots (*blue*, n = 212), and all spots (*black*, n = 6491) for all 14 MTCs. Significant spots occurring in multiple time bins (4 ms) were only counted once. **(C)** Receiver operator characteristics (ROC, see Materials and Methods) for detection of inhibitory spots (n = 14 MTCs). Curves of five MTCs (*yellow*) separated well from the diagonal (*gray*), ROC curves of the remaining nine MTCs (*black*) did not clearly separate from the diagonal.

We examined the spatial distribution of excitatory and inhibitory sub-threshold inputs to individual MTCs in more detail. For each stimulated spot, we calculated the resulting average membrane potential in 4 ms time bins. From this, we again subtracted the average membrane potential modulation (**Figure 7B**, *black trace*) and displayed the values for all spots jointly as a movie in false colors (representative example in **Figure 8A**). We observed a significant depolarization of the membrane potential directly at and close to the hotspot. The weak, however, obvious excitatory components around the hotspot is in accordance with the observation of subtle MTC activation by stimulation of the close surrounding area (compare to **Figures 5B,C**), which might be explained by stimulation of MTC dendrites. After a short depolarization, numerous spots that hyperpolarized the membrane potential emerged up to 500 μm away from the hotspot. This hyperpolarization was very short, i.e., occurring in a time window of <20 ms. Comparing the spatial distribution of significant membrane potential fluctuations from all recordings, we found a clear separation of excitatory and inhibitory spots: 90% of excitatory spots were closer than 130 μm (**Figure 8B**, *p* < 10^-20^, KS test, *n* = 14) to the hotspot. Spots that significantly hyperpolarized the membrane potential were distributed within 500 μm from the stimulation hotspot (spots were counted as excitatory or inhibitory, if their effects passed the significance threshold of α < 10^-3^ and had a magnitude >25% of the most effective excitatory or inhibitory spot).

Although, all MTCs recorded in whole-cell configuration had excitatory hotspots, we did not detect inhibitory spots in the surrounding area of all neurons. To identify these MTCs we calculated ROC curves for all MTCs (**Figure 8C**). In 4 ms time bins, we calculated the number of significant inhibitory spots before the stimulation of the respective spot (false positive rate) and between 0 and 40 ms after stimulation onset (true positive rate), varying the significance criterion. The resulting values were plotted against each other (**Figure 8C**, significance thresholds ranging from α = 10^-5^ to 1): in 5 out of 14 MTCs the ROC curves separated well from the line of identity, indicating detection of specific hyperpolarizing spots at the end or shortly after the stimulation.

### Distance-Dependent Inhibition

**FIGURE 9:**
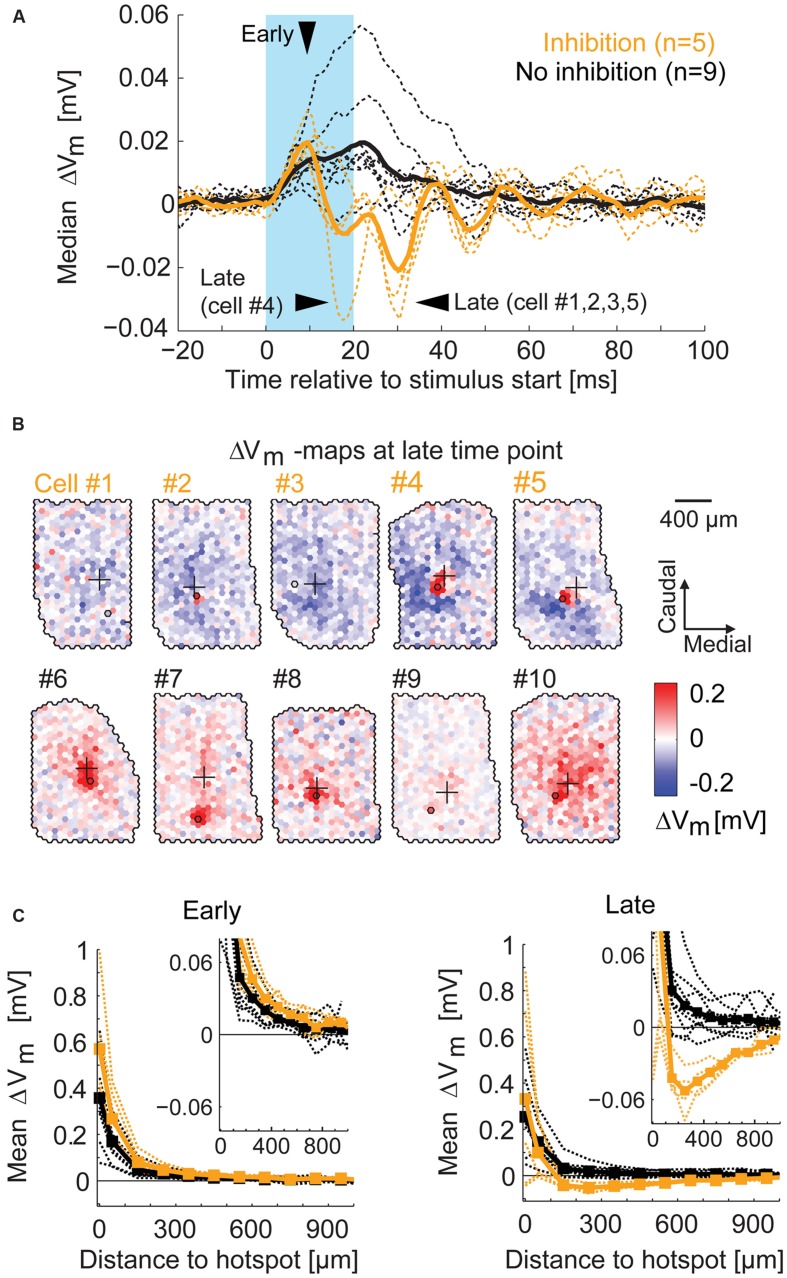
**Spatial profile of surround inhibition. (A)** For each MTC the median of membrane potential changes across all spots (*broken lines*) displays the general amount of inhibition. Arrowheads point to the time bins of maximal excitation (‘*early*’) and inhibition (‘*late*’). *Thick lines*, median across all MTCs with (*yellow*) and without (*black*) inhibition; *blue box*, stimulus. **(B)** Example maps of membrane potential change (4 ms time bin, ‘*late*’) for different MTCs among the two groups. *Yellow numbers*, MTCs with significant inhibitory spots at time of maximal inhibition; *Black numbers*, MTCs without significant inhibition at corresponding time points. **(C)** Membrane potential change as a function of distance from the hotspot (averaged over equidistant spots in 100 μm bins). *Broken lines*, individual MTCs; *thick lines*, average. *Left*, spatial profiles at the time point of maximal excitation (see **A** ‘*early*’). *Right*, spatial profiles at the time point of maximal inhibition (see **A** ‘*late*’). *Inset*, zoom-in revealing the peak of inhibition.

To visualize the time course of the overall excitatory and inhibitory components around stimulation, we calculated the median membrane potential change in every time bin across all spots of a given recording (**Figure 9A**). From the ROC classification, we were able to separate the two groups of MTCs with and without surround inhibition (*yellow* and *black* traces respectively). For the MTCs with surround inhibition, the membrane potential revealed the excitatory phase followed by a predominantly inhibitory phase. From these time courses we determined the time bin of maximal excitatory effects (4–8 ms after the stimulus start, **Figure 9A**, *’early’*), and the time bins of maximal inhibitory effects (18–33 ms after the stimulus start, **Figure 9A**, *’late’*). The membrane potential maps at the late time points are presented for all MTCs with surround inhibition (*yellow*) and for five representative MTCs without surround inhibition (*black*, **Figure 9B**). The maps of MTCs with surround inhibition showed spatially diverse inhibitory receptive fields, whereas only excitatory components were present in the remaining MTCs. To evaluate the distance dependence of the effects with respect to the hotspot, we generated spatial profiles of the different maps at early and late time points (profiles were centered on their hotspots and calculated from the average membrane potential change across equidistant spots; 100 μm bin width). The spatial profiles were nearly identical for all MTCs during the early period of excitation (**Figure 9C**, *left*). They differed, however, between the two groups during the phase of maximal inhibition (**Figure 9C**, *right*). In all five MTCs with surround inhibition, the average inhibition was strongest within 200–500 μm from the hotspot (**Figure 9C**, *right*).

Taken together, the maps that revealed inhibitory effects in the surrounding area were highly heterogeneous between different MTCs. However, with increasing distance from the glomerular unit we recorded from, the hyperpolarization induced by the optical stimulation dropped (**Figure 9C**). Our data support the notion that the strength of specific inhibitory interactions between MTCs is distance dependent and can extend up to 500 μm.

### Inhibition by Stimulation of the Surrounding Area is Fast and Phasic

**FIGURE 10:**
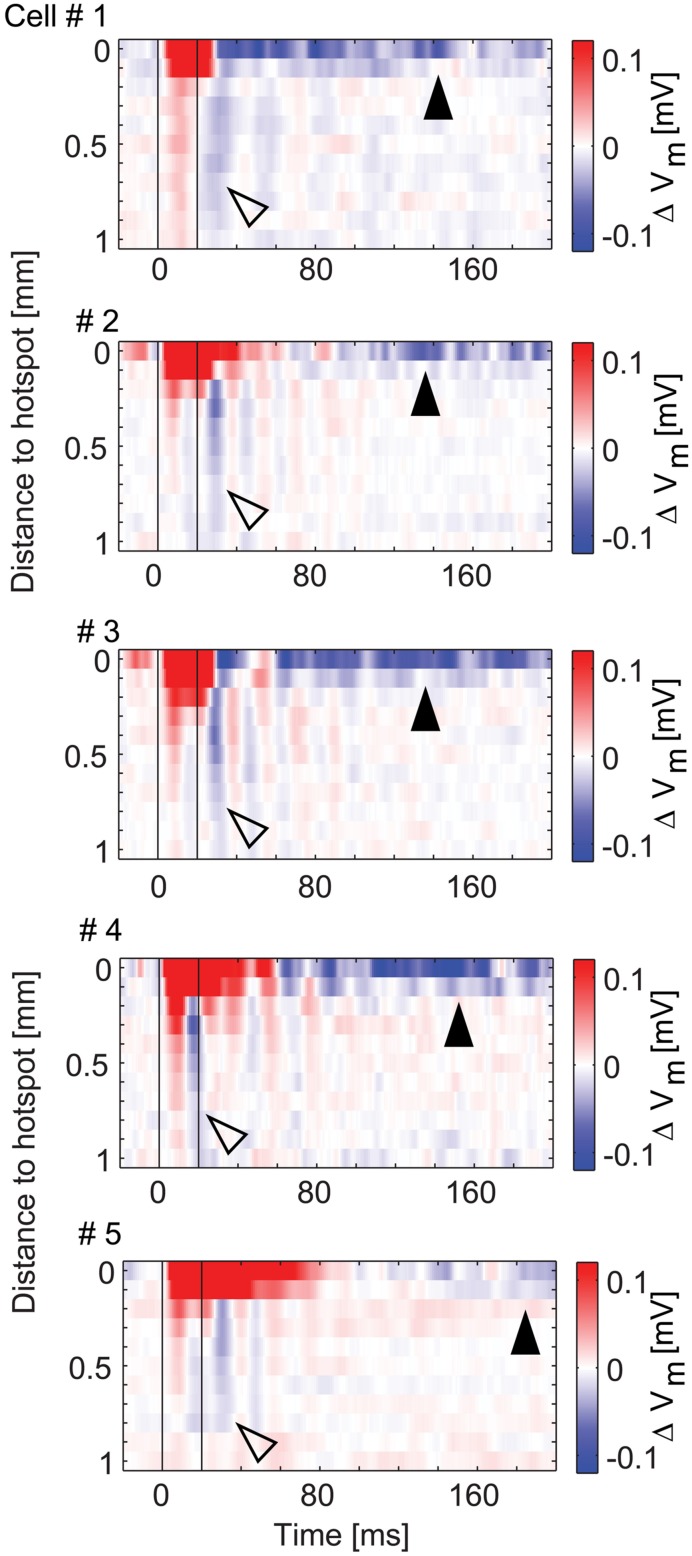
**Spatio-temporal profiles of excitatory and inhibitory effects.** Color-coded membrane potential change as a function of distance from hotspot (100 μm bins) and time (1 ms bins) for the five MTCs with inhibition. *Black lines*, begin and end of stimulus. Arrowheads point to inhibitory effects at the hotspot (*closed*) and in the surrounding area (*open*).

The data presented so far revealed complex spatial and temporal features of inhibitory effects on MTCs. For the five MTCs with surround inhibition, we therefore analyzed the spatial profiles evolving over time (**Figure 10**). To this end we color-coded the average membrane potential change at different distances from the hotspot (y-axis, 100 μm bins) and plotted it at different time points relative to the stimulus start (x-axis, 1 ms bins). The profiles show depolarizing effects around the hotspots during the stimulus, delayed and long lasting hyperpolarizing effects at the hotspots, as well as phasic hyperpolarizing effects in the surrounding area shortly after the stimulus: the most negative values at the hotspot occurred 60–200 ms after stimulus onset. On the contrary, hyperpolarization corresponding to surround-stimulation occurred mainly within a very short period of 15–40 ms after stimulus onset.

**FIGURE 11:**
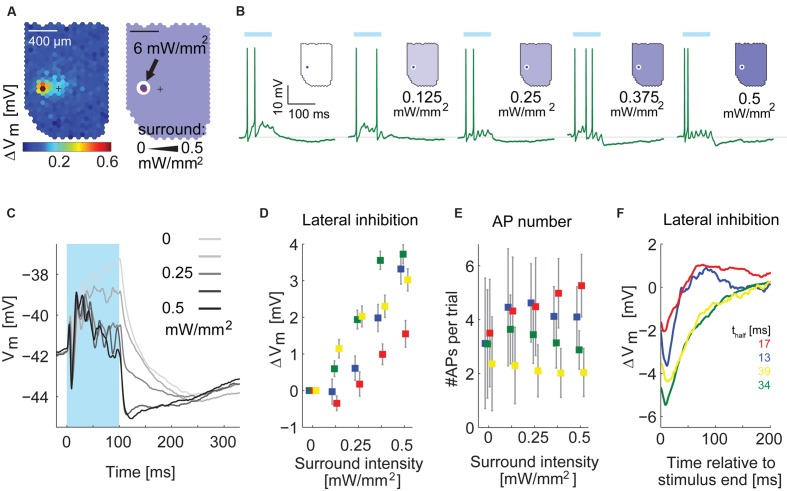
**Inhibition by surround stimulation is fast and phasic. (A)** *Left*, functional map with hotspot (*black hexagon*) and pipette tip position (*cross*). *Right*, schematic of the center-surround stimulus. Stimulation intensity was 6 mW/mm^2^ for the center and varied between 0 and 0.5 mW/mm^2^ for the surrounding area. **(B)** Single responses to stimulation of the hotspot in combination with varying intensities of the surround illumination, recorded in whole-cell configuration, *Blue bar*, 100 ms stimulus. **(C)** Membrane potential of the cell in **(A,B)** averaged across 50 trials for five different surround conditions. APs were removed before averaging. Different gray values indicate different intensities of the surround stimulus. In panels **(D–F)** this MTC is colored green. **(D)** Increase of inhibition by the surround stimulus as function of the surround intensity for four MTCs. Data points show the average difference (Mean ± SEM) between traces with surround stimulation and traces with exclusive center stimulation; colors correspond to single MTCs (same color code as in **E,F**). **(E)** Average number of APs (±SD) per trial as a function of intensity of the surround illumination for four MTCs. **(F)** Time courses of the difference between combined center and surround stimulation (*black trace* in **C**) and center stimulation alone (*lightest gray* in **C**) for four MTCs. *Bottom right*: t_half_ values give the time values from peak to half peak for the four displayed cells.

To confirm the timing differences, we applied a simpler stimulus in four MTCs. We flashed the hotspot of a given MTC for 100 ms at full intensity (diameter: *d* < 100 μm, 6 mW/mm^2^) in combination with moderate stimulation of the surrounding area (0–0.5 mW/mm^2^, *d* > 100 or *d* > 190 μm, representative example in **Figures 11A–C**). Illuminating only the hotspot caused the MTCs to depolarize and fire (**Figure 11B**). This was followed by hyperpolarization after the end of the stimulus. Increasing the stimulation intensity in the surrounding area, the post-stimulus hyperpolarization became stronger (example cell in **Figures 11B,C**; summary of membrane potential changes at different stimulus intensities of all recorded MTCs in **Figure 11D**). Notably, the number of evoked APs between the different conditions was stable: increasing the surround intensity hardly changed the overall AP rate of the recorded MTCs (**Figure 11E**). Thus the increase in inhibition could not be explained by recurrent inhibition that would get stronger with more APs elicited in the MTC itself.

To isolate the time course of hyperpolarization induced by surround stimulation, we subtracted the membrane potential time courses of exclusive hotspot stimulation from the data with maximal surround stimulation of 0.5 mW/mm^2^ (**Figure 11F**, *n* = 4 cells). The resulting traces had a faster time course (time from peak to half peak ranging from 13 to 40 ms) than the long lasting hyperpolarization after stimulation of the hotspot alone. In all four cells the slow hyperpolarization after exclusive hotspot stimulation never peaked earlier than 130 ms after stimulus end. Thus with a temporally and spatially much simpler stimulus than the white noise, we could confirm the finding that the hyperpolarization arising from surround stimulation is phasic and acts on a faster time scale than the slow hyperpolarization observed after hotspot stimulation.

## Discussion

In this study we examined the functional connectivity between MTCs in the mouse dorsal OB by using an optogenetic approach combined with electrophysiological recordings, in juxtacellular or whole-cell patch clamp configuration. We drove MTCs using light spots the size of single glomeruli, i.e., the functional unit of OB. In this setting, we investigated the effect of stimulating MTCs at varying distances to the MTC we recorded from. In Thy1-ChR2-YFP mice the majority of MTCs express channelrhodopsin2 (ChR2) while no other neurons in the OB express the light-sensitive ion channel (Arenkiel et al., 2007). Using these rather than OMP-ChR2 mice (Dhawale et al., 2010; Smear et al., 2011) we directly stimulated MTCs. Thereby we bypassed inhibitory mechanisms that are mediated in the glomerular layer during sensory input (Fukunaga et al., 2014), although we could not completely rule out the possibility that apical dendrites of stimulated MTCs activated juxtaglomerular cell types responsible for intraglomerular interactions (Murphy et al., 2005; Homma et al., 2013). However, by choosing this experimental setting we focused our analysis on the second layer of computations, i.e., the EPL, which is suggested to provide a network that mediates recurrent and lateral inhibition (Shepherd et al., 2004; Miyamichi et al., 2013).

The optical stimulus we applied revealed distance-dependent lateral inhibition at distances even beyond 500 μm (corresponding to approximately five glomerular diameters) from the parent glomeruli of the MTCs we recorded from. The functional maps that describe the spatial features of lateral inhibition were heterogeneous: they did not exhibit symmetry around the parent glomerulus of a given MTC, but still they contained distance-dependent characteristics. These findings functionally confirm anatomical results from a study of Kim et al. (2012) that described connectivity schemes with similar spatial characteristics. Furthermore, the lateral hyperpolarization we observed is restricted to a short time window, well in line with findings from Kato et al. (2013) and Fukunaga et al. (2014), suggesting that GC- as well as PV cell-mediated inhibition acts at fast time scales.

### Comparison with Studies Addressing Inhibition with Odor Stimuli

Our results are significant in the context of previous approaches studying inhibitory interactions using odor stimuli (Luo and Katz, 2001; Fantana et al., 2008). These studies yielded conflicting results. Calculating response correlation maps between intracellular recordings and intrinsic signal imaging maps in mice, Luo and Katz (2001) described a dynamic center-surround organization. Our optogenetic approach was able to overcome two major limitations of this study: (1) Performing intracellular recordings, Luo and Katz (2001) had to limit their stimulus set to 4–6 odorants per experiment; the low number of stimuli did not allow for exclusive or independent stimulation of different glomerular units since the odors used in their study activated multiple glomeruli at once. This co-activation might have led to biased and overestimated response correlation maps. In other words, the effects of near and distant co-activated glomeruli could not be differentiated. (2) Using intrinsic signal imaging Luo and Katz (2001) treated the input to the OB as static patterns. However, calcium imaging experiments have since demonstrated that the input from the OSNs is not static but evolves over time with odorant-specific dynamic patterns (Spors et al., 2006; Carey et al., 2009). Our DMD based projection system typically projected 15,000–30,000 different stimulus patterns per experiment with sub-millisecond precision, leading to depolarization and an AP rate increase in MTCs within a few milliseconds. Furthermore, the combination of glomerular units flashed in a given stimulus frame was random. By this, we did not repeatedly co-activate an identical set of glomerular units, but instead activated different units independently from each other.

In the other approach to estimate functional connectivity *in vivo*, Fantana et al. (2008) could not rely on the sensitivity of intracellular recordings since they performed long-lasting experiments with a large odorant set (up to 100 odors). They compared input patterns, imaged as intrinsic optical signals, with AP output recorded extracellularly from MTCs from different rats. Least square regression avoided overestimating the receptive field size. Their findings supported two notions: (1) inhibitory receptive fields (in terms of the glomeruli influencing a given MTC) are sparse, and (2) lateral inhibitory connections are distance-independent. In contrast, we found strong evidence for distance-dependent inhibitory interactions between MTCs. However, the spatial characteristics we observed were only resolvable by performing whole-cell patch clamp recordings. The analysis of AP responses alone did not reveal detailed results on the spatial arrangement of inhibitory interactions. Considering results from Fukunaga et al. (2014), it becomes obvious that interactions on fast timescales can only be examined by having precise temporal control on the stimulus as well as having sufficient resolution in recordings of the neuronal response. Therefore our experimental setting, with the temporal precision of the artificial stimulation and the analysis of membrane potential changes, allowed us to reveal interactions that might not have been resolved by the previous studies.

### Limitations of Experimental Setting

In this study, we used artificial stimuli (optical generated patterns). Therefore, the layout of MTC activation in our paradigm may differ from the activation based on naturally occurring odorant stimuli. It has been shown, that most of odorants in naturally occurring concentrations activate approximately 10–30% of all glomeruli on the dorsal OB (Vincis et al., 2012). Based on this finding, the spatial characteristics of the stimulus we used (10% of the OB surface illuminated at a given time) are a reasonable approximation to naturally occurring activation patterns. However, we could not exclude that stimulating multiple spots on the OB surface in fast sequence might have led to an unnaturally high activation of MTCs. As a consequence, the tone of inhibition mediated by GCs and PV-cells in the EPL network might differ to the natural conditions. This could be an explanation for our finding of only subtle and non-specific lateral inhibitory influence on the AP rate of MTCs. Thus using an artificial stimulus, we cannot rule out that we missed lateral inhibitory effects shaping the output of MTCs under natural conditions. In future studies, the creation of more realistic MTC activity patterns by optical stimulation might be a way to simulate natural conditions in order to put the findings in a more behaviorally relevant context.

Furthermore, experiments in this study were performed on animals anesthetized with Urethane. Several studies have shown that spontaneous MTC activity as well as odorant-evoked MTC activity differs between awake and anesthetized states (Rinberg et al., 2006b; Kato et al., 2012). Diverse effects have been observed on neuronal activity in the OB, depending on the anesthetics used and on the experimental readout (also see Lin et al., 2006; Vincis et al., 2012). So far, there is no comprehensive understanding of the effects that anesthetics have on neuronal activity in the OB network. Nevertheless, we cannot exclude that Urethane anesthesia influenced the extent and dynamics of inhibitory effects between MTCs that we described here. Investigating spatio-temporal characteristics of lateral inhibition with our approach in awake animals will be important in order to verify behavioral relevance of our findings.

### Source of the Oscillations in the Gamma Band

In our experiments, prolonged full field optical stimulation (>0.5 mW/mm^2^) evoked a γ-oscillation and illumination with dense noise stimuli (∼10% of the surface illuminated @ 6 mW/mm2, 50 Hz) induced a pronounced oscillation locked to the frame transition rate within the gamma band. The MTC/GC network has a tendency to oscillate *in vivo* (e.g., Adrian, 1950; Freeman, 1972) and *in vitro* (Lagier et al., 2004). *In vivo* this γ-oscillation is typically strongest at the same time of the strongest sensory input to the OB (Rojas-Libano and Kay, 2008). Our data are consistent with the argument that γ-oscillations primarily depend on activation of MTCs no matter if this is due to sensory stimulation, antidromic stimulation or direct optical stimulation, as in our case (Shepherd et al., 2004). Most likely, however, the oscillatory activity in our experimental setting is not only a natural phenomenon arising from interactions in the OB circuitry. Presumably, the rhythmicity of the noise stimulus (activating a great number of MTCs at once) imposed part of the oscillatory activity and locked the LFP and AP activity tightly to the frame transition frequency. Since the focus of our analysis did not lie on inhibitory effects that generate the oscillatory activity but on spatial characteristics of inhibitory interactions, we subtracted the oscillations. However, it is noteworthy that the spatial inhibitory interactions observed in this study were nested in oscillatory activity that is also naturally occurring as well as functionally and behaviorally relevant.

### Inhibitory Mechanisms at Different Timescales

Our results revealed clear differences in the time course of two inhibitory effects: stimulation of the parent glomerulus of a given MTC resulted in slow and long-lasting inhibitory effects, whereas stimulation of spots in the surrounding area yielded inhibition on a fast timescale. After stimulation of the hotspot, we observed a significant relationship between the overall activity level of individual neurons and the strength of slow inhibition. This could be explained by the findings of Margrie et al. (2001), who showed that bursts of APs propagate more reliably and further along MTC lateral dendrites than single APs. Therefore, they might activate more inhibitory interneurons resulting in stronger recurrent inhibition. An alternative explanation for the same observation could be a floor effect: since the AP rate cannot be negative, MTCs with low baseline activity can only have small reductions in AP rate. Moreover, voltage gated intrinsic conductances of MTCs could contribute to this observation but were not investigated here. Having a timescale compatible with a slow and long-lasting inhibition, an alternative mechanism causing long lasting recurrent inhibition could be the intra-glomerular recurrent inhibition mediated by periglomerular cells (Murphy et al., 2005; Fukunaga et al., 2014).

It is important to note that with our optical stimulation method, we jointly stimulated MTCs belonging to the same glomerulus. Considering also that MTCs can activate each other via glutamate spillover and MTCs of the same parent glomerulus can have inhibitory connections (Urban and Sakmann, 2002), we could not distinguish recurrent inhibition from lateral inhibition between sister MTCs belonging to the same glomerulus. However, the very diverse time courses of inhibition observed, following the surround stimulation and the parent glomerulus illumination, suggest two different mechanisms.

### Alternative Explanations

We assumed that the most likely mechanism for lateral inhibitory effects observed in this study is that APs generated by illumination of the MTC glomerular tuft propagated into lateral dendrites. The glutamate release onto GC or PV cells then would result in lateral and recurrent inhibition. However, different pathways of inhibitory interactions could also explain our data: (1) Direct light-induced depolarization of MTC lateral dendrites could result in recurrent inhibition via inhibitory interneurons. However, this mechanism is unlikely since lateral dendrites were illuminated to a lesser extent than the glomerular tufts due to their deeper location. In addition, AP propagation in the lateral dendrite of a neighboring MTC is more efficient than pure electrotonic conduction of inhibitory post-synaptic potentials in the recorded MTC. IPSPs elicited far away from the soma of a recorded MTC (after stimulation of its lateral dendrite) would experience a significant amplitude drop during their propagation to the soma. Thus, it is more likely that we detected IPSPs that were elicited close to the soma by APs of neighboring MTCs that traveled in the lateral dendrites of these neighboring MTCs. Also, if one assumes that the observed surround inhibition is a result of precedent activation of lateral dendrites at these locations, then excitatory and inhibitory components in the maps would cover the same areas (compare to **Figure 8A**). Thus, the dissimilar spatial profiles of excitation and inhibition we observed point toward a different mechanism. (2) Feedback from activated cortical neurons could drive GCs at their somata and indirectly result in MTC inhibition. However, data from another study (Laaris et al., 2007) show that antidromic MTC activation via the LOT mainly activates dendrodendritic synapses in the EPL. Therefore, a dominant effect by stimulation of the GCs at their cell bodies through feedback from cortical neurons seems unlikely. (3) Direct optical activation of axons from cortical pyramidal cells expressing ChR2 could drive GCs and in turn inhibit MTCs, but since the fraction of cortical neurons expressing ChR2 in the Thy1-ChR2-YFP mouse line is small compared to the fraction of MTCs (Arenkiel et al., 2007), this last mechanism seems unlikely too.

### Two Groups of MTCs

An important additional finding of our study is that we did not identify lateral inhibitory influence on all MTCs: a large proportion of recorded cells did not show inhibitory receptive fields at all. This could be explained by different integration of MTCs in the inhibitory network of the EPL (Schneider and Scott, 1983). The functional differences we describe here could represent the two distinct populations of MTCs, with mitral cells receiving surround inhibition and tufted cells not. Furthermore, within the population of mitral cells there are diverse morphologies (Orona et al., 1984; Ezeh et al., 1993), thus one would most likely also see diverse inhibitory receptive fields. Mitral cells with lateral dendrites branching in different laminae of the EPL also suggest distinct functional integration in the inhibitory network, and could result in differences in the inhibitory receptive fields. Therefore, future studies should address this issue by labeling recorded cells followed by morphological reconstruction. The anatomical identity of the recorded neurons together with their functional data might reveal valuable insights to the structure and scale of distinct inhibitory circuits that shape the output of MTCs.

### Spatial Profile of Inhibitory Maps

Our results suggest that inhibitory interactions between MTCs are neither *only distance dependent* nor *only sparse and specific.* There are studies reporting no chemotopy on a fine scale in the OB (Soucy et al., 2009); however, several other studies show that glomerular position is predicted by odorant receptor classes (Bozza et al., 2009; Pacifico et al., 2012) and that there are clusters of glomeruli activated by distinct substance categories (Rubin and Katz, 1999; Johnson and Leon, 2007). In addition, Ma et al. (2012) showed a correlation between activation profiles and distances between glomeruli. The data presented here also match anatomical results from Kim et al. (2012) and could point toward a restriction of lateral inhibitory interactions to functionally defined domains. In such a scenario, the area covered by a functional cluster would in turn predict the extent and shape of the inhibitory receptive field of a given MTC.

Neuronal architecture favoring distance-dependent inhibition does not prove that this organization is used for physiological function (Cleland et al., 2007). An important step for future experiments will be to determine glomerular locations as well as identify their molecular receptive ranges before functional mapping experiments. This would allow analyzing if molecular and anatomical clusters (Ma et al., 2012) are superimposed to functional areas of inhibitory interactions. Specific stimulation of morphologically outlined glomeruli could reduce the number of stimulation spots from 522 down to 70 to 100, in the 2 mm^2^ field of view, and substantially facilitate the computation of higher order correlations and allow the examination of activity-dependent changes of connectivity (Arevian et al., 2008). Comparing molecular receptive ranges between glomeruli that do and glomeruli that do not inhibit specific MTCs will most likely provide a deeper insight into the logic of functional connections within the dense EPL network.

## Author Contributions

HS and AL designed the study. MV and HS built the optical stimulation setup including the stimulation and data acquisition software. AL performed the experiments. AL and HS analyzed the data. AL, AD, and HS wrote the manuscript.

## Conflict of Interest Statement

The authors declare that the research was conducted in the absence of any commercial or financial relationships that could be construed as a potential conflict of interest.
